# The current state of forensic imaging – perspectives

**DOI:** 10.1007/s00414-025-03466-6

**Published:** 2025-03-21

**Authors:** Fabrice Dedouit, Mathilde Ducloyer, Jamie Elifritz, Natalie L. Adolphi, Grace Wong Yi-Li, Summer Decker, Jonathan Ford, Yanko Kolev, Michael Thali

**Affiliations:** 1 Department of Forensic Pathology, Bâtiment Raymonde Fournet, Place du Dr Baylac, Hôpital Purpan, Toulouse, 31700 France; 2 Department of Forensic Pathology, Nantes University, University Hospital, Bd Jean Monnet, Nantes, F-44000 France; 3 Forensic Radiology Group, Anderson, SC USA; 4 Office of the Medical Investigator, University of New Mexico, Albuquerque, NM 87131 USA; 5 Department of Radiology, Penang General Hospital, Jalan Residensi, Georgetown, Penang 10450 Malaysia; 6 Departments of Radiology and Pathology, University of Southern California Keck School of Medicine, 1450 San Pablo Street, Suite 3500, Los Angeles, CA 90033 USA; 7 Department of General Medicine, Forensic Medicine and Deontology, Medical University-Pleven, 1 St Kliment Ohridski str, Pleven, 5800 Bulgaria; 8 University Zurich, Virtopsy Group, Switzerland

**Keywords:** Forensic imaging, Forensic science, Photogrammetry, 3D surface scanning, 3D printing

## Abstract

This fourth part of the review of the current state of forensic imaging describes the future potential influence of artificial intelligence in forensic imaging. In addition to this important point, training in forensic imaging is discussed in detail, as are the documentation possibilities offered by non-conventional imaging tools such as photography, photogrammetry, 3D surface scanning and 3D print casts.

## Introduction

**Fig. 1:**
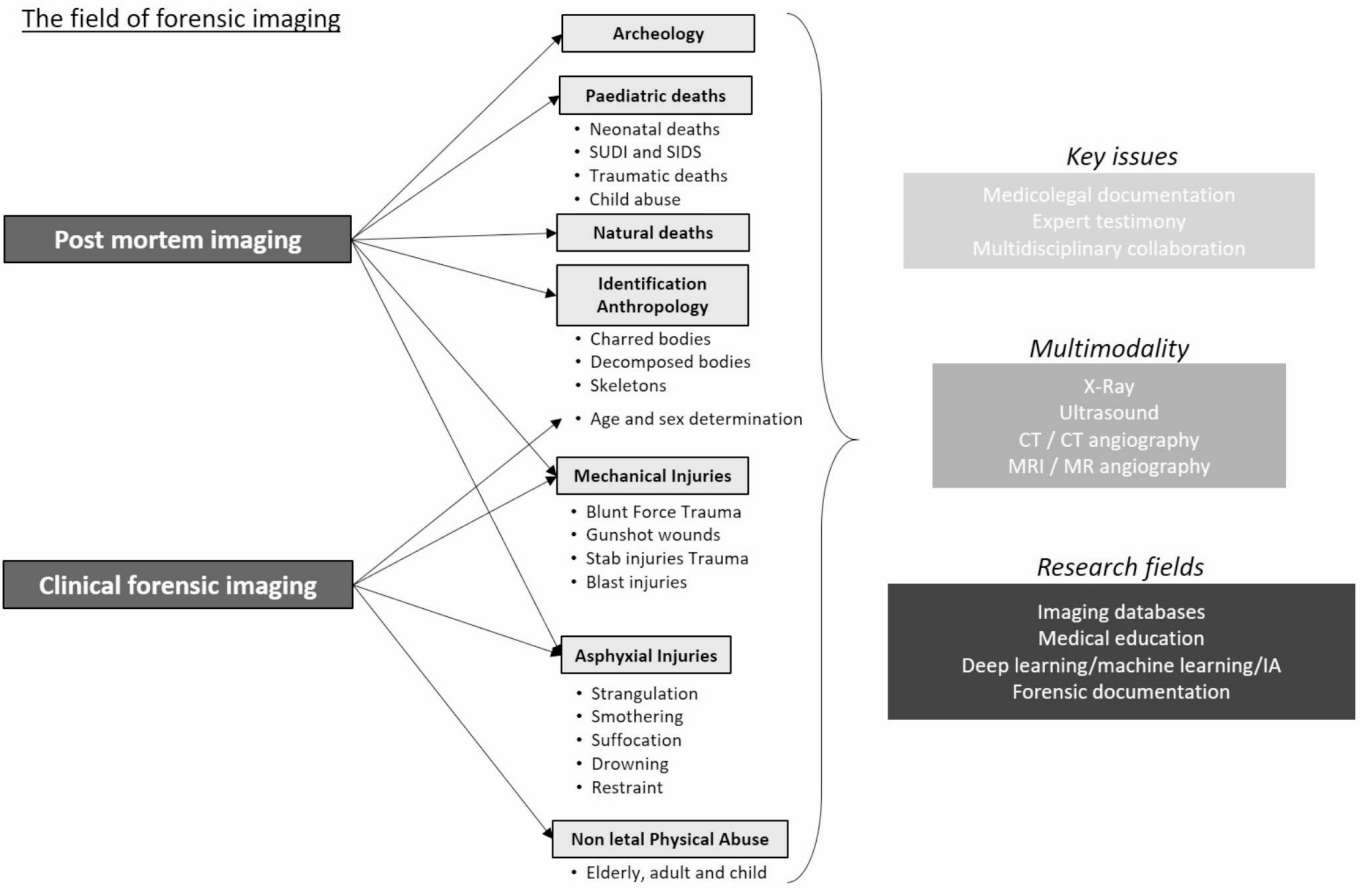
Illustration of the main indications and benefits of post-mortem imaging in the most common forensic situations

Forensic imaging is growing worldwide. As it follows the trend of using and adapting classical radiological tools, the introduction of artificial intelligence seems obvious. This article presents different possibilities of deep learning and machine learning with potential applications in forensic and anthropological imaging. In this way, the creation of international databases dealing with forensic examinations seems crucial. These questions about database creation dovetail perfectly with another important issue for the future of forensic imaging: education. Education is obviously the key to the future, the diffusion and the growth of forensic imaging. This article will also explore the range of imaging tools available for creating images that are not secondary to X-rays, magnetic resonance, or ultrasound, including photography, photogrammetry, 3D surface scanning, and 3D printing of casts. As illustrated in Fig. 1, these perspectives are interconnected with contemporary issues in the domains of post mortem and clinical forensic imaging.

This fourth and final section of the review of the current state of forensic imaging outlines the various pathways that could be pursued by forensic radiologists and researchers in the coming years.

## Most promising perspectives in forensic imaging

### Imaging databases

The widespread use of post-mortem imaging has generated a large volume of imaging data. While each centre can organize a database locally, there is a need to create and coordinate large-scale post-mortem imaging databases. This is the only way we will be able to pursue preliminary works on particularly rare cases or paediatric cases, by increasing the number of cases for research questions, homogenizing data and limiting the effects of high variability in forensic cases. To date, there is relatively little literature on the subject. We can cite as examples the New Mexico Decedent database, which is without doubt the biggest data repository of postmortem imaging that is accessible to any research team [1]. The Netherlands also has a large database, but access to it is currently limited to Dutch researchers [2]. A Swiss team has proposed a “centralized virtopsy database”, as an online tool for associating imaging with autopsy findings [3]. Other applications of this type need to be developed and disseminated. Several obstacles still need to be overcome before progress can be made on this issue: the need for agreements between research teams on the purpose and methods of these imaging databases, the content of the imaging databases and the associated clinical data, compliance with regulatory and legal requirements, which vary from country to country, and respect for essential ethical considerations. It also implies obtaining private or public funding to cover the cost of image and data storage, storage security and human time to implement the database prospectively.

### Artificial intelligence and deep learning

#### Definitions

It is often said that the difference between machine learning and deep learning is that machine learning algorithms process quantitative and structured data (numerical values), whereas deep learning algorithms process unstructured data such as sound, text or images.

**Machine learning** is a branch of artificial intelligence concerned with the development and study of statistical algorithms that can effectively generalize to perform tasks without explicit instruction. Supervised learning algorithms build a mathematical model of a set of data containing both the inputs and the desired outputs. The data is called training data and consists of a set of training examples. Each training example has one or more inputs and the desired output, also known as the control signal. By iteratively optimizing an objective function, supervised learning algorithms learn a function that can be used to predict the output associated with new inputs. Machine learning can involve creating a model that is trained on some training data and can then process additional data to make predictions. Different types of models have been used and researched for machine learning systems (for example, artificial neural networks (ANNs), or connectionist systems, are computational systems vaguely inspired by biological neural networks).

Support vector machines (SVMs), also known as support vector networks, are a set of related supervised learning methods used for classification and regression.

Typically, machine learning models require a large amount of reliable data for the models to make accurate predictions. When training a machine learning model, machine learning engineers need to target and collect a large and representative sample of data.

**Deep learning** is the subset of machine learning methods based on artificial neural networks with representational learning. The adjective “deep” in deep learning refers to the use of multiple layers in the network. The methods used can be either supervised, semi-supervised or unsupervised. Deep learning architectures such as deep neural networks, deep belief networks, deep reinforcement learning, recurrent neural networks, convolutional neural networks, and transformers have been applied to fields such as computer vision, speech recognition, natural language processing, machine translation, bioinformatics, drug design, medical image analysis, climate science, material inspection, and board game programs, where they have produced results comparable to, and in some cases surpassing, human expert performance.

#### Applications in anthropology and forensic medicine

**Machine learning techniques and data mining algorithms** are essential tools for exploring, extracting and explaining data. The results of these techniques allow us to identify the patterns in the data and build models to predict future behaviour in a specific context. A predictive model can be learned from different individuals and a set of heterogeneous data. This model could then be used to accurately predict the outcome of new samples. Data mining will also have a positive impact on the reconstruction of the human biological profile by enabling informed decision making. Previous studies have investigated the application of machine learning and data mining to medical records. Previous studies have applied data mining and machine learning methods to sex estimation using bone measurements. Several machine learning methods have been published using different algorithms: k-nearest neighbour, Gaussian naive Bayes, logistic regression, decision tree, linear discriminant analysis and support vector machine). All these algorithms can be applied to the dataset to obtain a predictive model and generate prediction rules. In most articles, the performance of different algorithms is compared and the best one is retained [4].

Recent advances in deep learning (DL) have brought significant benefits to medical image analysis in various areas, such as liver cancer segmentation, pneumothorax segmentation, skin lesion analysis for melanoma detection, and autonomous cancer detection and classification. Some work has also been published on the prediction of drowning or fatal hypothermia on PMCT using DL [5, 6]. ]. However, DL is complex to understand and implement because of the large amount of data required for the training part. Different deep learning models, such as a convolutional neural network (CNN) and a densely connected convolutional network (DenseNet), can be used and trained. For all algorithms, a large percentage (between 70 and 80% of the data) is randomly selected for training, and the remaining percentage is used for validation. Few articles deal with age assessment, and DL is still a work in progress, but shows potentially incredible results. In one article, the authors showed that combining the PMCT scans of the femur and mandible as input to a 3D CNN could produce a more accurate age estimate for an adult [7]. Using a large dataset, they demonstrated the advantages of a DL-based method over traditional approaches in terms of speed, accuracy and reliability. An automated system is important to help experts determine a person’s chronological age based on their femur and mandible. These results demonstrate the potential of DL and encourage further research into its use in age estimation systems.

The democratization of radiology has led to a number of improvements, including the collection of larger databases for use in new studies, which ultimately increases the statistical power of the results, and the possibility of using artificial intelligence in radiology and forensic radiology because of the amount of data required.

Deep learning is a subfield of machine learning related to performance: it remains very complex to explain how an algorithm arrives at a result. One way is to “cut” a layer of your algorithm in the middle to study the output. But it’s not as easy as with traditional machine learning algorithms to know exactly how a deep learning algorithm works. So there could be legal constraints. In certain fields (finance, banking, etc.), professionals are obliged to ensure that the algorithm can be explained: it is therefore restrictive to implement such algorithms.

In terms of the specific computational approaches followed by the automated methods, most studies rely on fully automated and deep learning-based solutions, which leads to two important improvements. As with any end-to-end method, the dataset only needs to be annotated with the expected output, i.e. age, reducing the time required for this process and thus allowing larger datasets of thousands of images to be assembled. Furthermore, these methods do not rely on specific bone structures specified by an expert, but rather on the parts of the image that the algorithm considers most relevant for the task at hand, and can work even if some parts are missing. Although these automatic methods have been shown to improve the performance and applicability of age estimation methods, their validation needs to be improved. The relative newness of deep learning techniques means that no automatic method has been tested on populations or collection devices other than the original ones, raising doubts about their generalization to different scenarios [8].

#### Use of finite element analysis in forensic medicine

With recent developments in advanced medical imaging and in silico technologies, non-invasive techniques such as CT-based finite element analysis have been widely used to study bone strength in adults, particularly in the application of fall prediction [9–12]. CT-based finite element models of pediatric femora have been developed and simulated under bending and torsional loading to provide personalized information on bone strength for infants and very young children during the rapid growth phase. The current study suggests that the finite element approach, which has been widely and successfully used in adults, can be adapted and applied to children. As pediatric bone samples are very difficult to obtain, the use of non-invasive techniques such as CT-based finite element analysis provides a valuable alternative for investigating pediatric bone biomechanics. In the future, this technique will allow us to create surrogate models for infants and very young children and provide more quantitative information on bone growth and strength to dramatically improve the limited information currently available in the literature [13]. Other applications have been proposed, like head and face trauma, in adult and children, and gunshot trauma [14–17].

### Education

With the widespread use of post-mortem imaging and the increasing number of forensic pathologists and radiologists aware of this issue, training is becoming more important than ever. At the same time, the use of post-mortem imaging as a teaching aid must also be seen as a means of improving the quality of education.

In recent years, several well-referenced and illustrated educational books have been published, detailing how to perform and interpret post-mortem imaging, based on the latest scientific findings and the authors’ own experience [18–23].

Face-to-face training opportunities remain relatively limited and are often unevenly distributed across the country. University training courses offering theoretical courses combined with console-based reading exercises are still the best way to get to grips with forensic imaging in real-life conditions. The main obstacle to accessing these certification courses is their generally high cost (registration, travel, accommodation) and limited number of places. Distance learning, made possible by the pandemic, should be considered as a serious alternative. While this teaching method has ensured educational continuity, the rapid development of innovative teaching aids has paved the way for new teaching methods, accessible to the widest possible audience [24]. Building high-quality, gradual, certifying distance learning courses is one of the challenges facing forensic imaging teachers and researchers in the coming years. Last, we can cite the ISFRI congress which takes place annually all around the world, as an incredible way to make the international community of forensic radiologists and pathologists bigger and connected and of course to present the latest updates and research on forensic imaging.

Post-mortem imaging must also be seen as a genuine teaching tool, especially for anatomy. Several anatomy teaching programs have integrated post-mortem imaging, enhanced or not, into their curriculum. PMCT and PMCTA in adjunction to conventional support like cadaver dissection, have proved their interest in the understanding of head, neck and vascular gross anatomy, as in osteology [25–27]. The addition of PMCT to traditional gross dissection is a powerful way to expose medical students to cross sectional imaging, it can improve their ability to better distinguish anatomical structures on CT, and it offers to them a better understanding of spatial anatomical relationships [28]. Last, we must cite the Dutch Fetal Biobank and Bernadette de Bakker’s works, which created an atlas of embryology from a bank of foetuses donated to science as a perfect example of how research, innovation and education can complement each other [29]. This atlas is not only an incredible way to revisit knowledge on embryology, it is also an effective and relevant educational tool for teaching and learning embryology [30].

### Forensic documentation

#### Surface scanning

Surface scanning technology has seen significant advancements in recent years, finding applications across various fields, including medicine, archaeology, and manufacturing. One of the most promising areas of application is in forensics. Surface scanning technologies, such as 3D laser scanning, structured light scanning, and photogrammetry, offer a range of capabilities that can greatly enhance forensic investigations, from crime scene documentation, evidence capture and preservation, victim documentation as well as any analyses derived from these captured data [31].

3D laser scanning is one method of capture that uses laser beams to acquire the shape of objects with high precision. The scanner emits a laser that sweeps over the surface of an object or scene, recording the time it takes for the laser to return to the sensor. This time-of-flight measurement allows the creation of a detailed 3D model of the object or scene. This technology is highly effective for scanning large areas and complex surfaces, providing detailed and accurate 3D representations. Some scanners are operated by hand and free to move in all directions covering very large areas or objects. Other scanners are designed for smaller objects and are stationary or mounted to a tripod, sometimes in conjunction with an automatic spinning platform.

Structured light scanners are similar in operation and appearance to 3D laser scanners, but just use a different mechanism for data capture. Instead of lasers, structured light scanning projects a series of light patterns onto an object and captures the deformation of these patterns using cameras. The deformation of the light patterns is analysed to determine the shape of the object. This method is faster than laser scanning and is particularly useful for capturing fine details and textures.

There is a significant difference between laser scanning and structured light scanning. Laser scanning produces a colourless 3D representation of the object, known as a “point cloud”, where every point on the scanned surface has precise, measurable spatial coordinates. Structured light scans, also photogrammetry 3D models, capture all the texture details and colours of the object. However, they require calibration and scaling for accurate measurements.

Currently, hybrid scanners, which combine laser scanning with structured light or photogrammetry, are becoming increasingly advanced. These scanners provide highly accurate surface relief details (from the laser) and detailed textures and patterns of objects (from the photographic data).

#### Photogrammetry

Photogrammetry involves taking multiple photographs of an object from different angles and using software to create a 3D model. This technique relies on identifying common points in different images to reconstruct the object in three dimensions. Photogrammetry is a cost-effective method and can be used with standard digital cameras. It is especially useful in outdoor environments and for large scenes where setting up scanning equipment may be challenging. It is possible to use drone mounted cameras to completely capture a scene with its surrounding environment [32]. Nearly any camera device can be a source for photogrammetry as long as a sufficient number of images are taken with a high enough resolution. Additionally, smartphone apps are available which allow for immediate on the fly scene or object capture which is most useful for in the field documentation.

#### Forensic applications of surface scanning and photogrammetry

Virtual technologies such as surface scanning and photogrammetry allow for the precise documentation of crime scenes. Traditional methods, such as sketches and photographs of the scene, are limited in their ability to capture the spatial relationships and exact dimensions of a scene. 3D scanning provides a comprehensive and accurate digital record that can be revisited and analyzed long after the scene has been cleared. This is particularly valuable in complex scenes where the positions of objects and evidence are critical. For instance, surface scanning and photogrammetry can capture the layout of a crime scene, including the positions of furniture, bloodstains, and other physical evidence. Once imported into a computer, these can be used to create virtual walkthroughs of the scene, aiding investigators in understanding the events that occurred and presenting the scene to juries in a clear and understandable manner [33–35].

Both photogrammetry and surface scanning provide a non-contact method to create detailed digital replicas of physical evidence, preserving it in its original state. This is particularly useful for fragile or perishable items, such as footprints in soil or snow, tool marks, and bloodstain patterns. For example, structured light scanning can capture the intricate details of a shoe print in mud, which can then be analyzed without the risk of damaging the original print. This digital preservation allows for repeated examinations and comparisons, ensuring that the evidence remains available for analysis even if the physical item deteriorates [36, 37].

3D models created from either method can be used to reconstruct events. For example, in traffic accident investigations, the scene can be accurately captured including the positions and conditions of vehicles, skid marks and debris, enabling precise reconstructions of the events leading to the collision. In forensic ballistics, 3D reconstructed scenes and victims can be used to analyze bullet trajectories and impact angles, helping investigators to determine the shooter’s position and the sequence of shots fired [38, 39].

In cases where the identity of a victim is unknown, digital capture of a skull can assist in facial reconstruction: the precise shape of a skull can be used by forensic artists and anthropologists to create a digital or physical reconstruction of the person’s face, aiding in identification efforts [40, 41].

Surface scanning and high quality photosets for photogrammetry can capture the minute details of tool marks left on various surfaces, such as metal, wood, or bone. These high-resolution scans can be analyzed to determine the type of tool used, its angle of impact, and other characteristics. Tool mark analysis using surface scanning can differentiate between marks made by different tools and even identify specific tools that were used in a crime [42–44].

With smartphone applications, first responders can arrive to a scene and capture critical information prior to a scene being disturbed for processing. Additionally, digital models can be easily shared and reproduced, enabling collaborative analysis and review by multiple experts. This reproducibility ensures that evidence can be examined by different parties without the risk of alteration or damage.

There are some limitations to this technology. High-end surface scanning equipment and software can be expensive, limiting accessibility for some forensic departments. Proper use of scanning technology requires specialized training, and the interpretation of scan data may require expert knowledge. Additionally, the large volume of data generated by surface scanning requires efficient storage, management, and processing capabilities. Handling and analyzing this data can be resource-intensive and may require specialized software and hardware. Digital data capture of a scene or object can be affected by environmental conditions, such as lighting, weather, and surface reflectivity. Adverse conditions can complicate the scanning process and reduce the quality of the data collected. For example, photogrammetry has severe limitations when it comes to reflective surfaces [45].

#### 3D printing

Once 3D data has been captured, there are several avenues open regarding what can be done with it. High quality visualizations can be created. Animations can be reconstructed. Simulations and analyses can be performed. Virtual reconstructions of crime scenes can be created in conjunction with augmented or virtual reality [33]. One other technology that is increasing in utilization for 3D data is the forensic application of 3D printing [46].

There are several methodologies that fall under the umbrella of 3D Printing.

Fused Deposition Modeling (FDM) is one of the most common and accessible 3D printing technologies. It works by extruding melted thermoplastic filament through a heated nozzle, which deposits the material layer by layer to build up the object. Common materials include PLA (polylactic acid), ABS (acrylonitrile butadiene styrene), PETG (polyethylene terephthalate glycol), and various composites. FDM printing is often the most affordable 3D printing technology.

Another 3D printing method is stereolithography (SLA) which uses a laser to cure liquid resin into hardened plastic. The laser selectively cures the resin layer by layer to form the object. The materials used are photoreactive-polymer resins, which can be formulated for various properties such as rigidity, flexibility, or high temperature resistance. This technology can be used to create high-detail prototypes, dental models, jewelry, and parts requiring smooth surfaces and fine details. Similar to SLA, Digital Light Processing (DLP) uses a digital light projector to cure liquid resin, but cures an entire layer at once using a digital projection of the layer’s cross-section.

Selective Laser Sintering (SLS) uses a laser to sinter powdered material, fusing the particles together to form a solid structure. The laser traces each cross-section of the object in the powder bed, layer by layer. SLS uses thermoplastic powders such as nylon, polyamide, and TPU (thermoplastic polyurethane) which are all good for complex geometries, and parts requiring high strength and durability. Surfaces of parts printed with SLS technologies often have a rougher texture that parts printed in SLA or DLP.

PolyJet technology jets layers of photopolymer resin onto a build platform and cures them with UV light. Similar to a common inkjet printer, multiple materials can be jetted simultaneously, allowing for multi-material and multi-color printing. PolyJet printers allow for high-detail prototypes with multi-material parts with multiple colors and rigidities. There are several other 3D printing technologies (such as Multi Jet Fusion, Binder Jetting, Direct Metal Laser Sintering, Selective Laser Melting, and Electron Beam Melting) but have limited practical forensic use.

#### Forensic applications of 3D printing

As a tactile form of visualization, 3D printing can be used in crime scene reconstruction. Traditional methods of documenting crime scenes (photographs, visualizations, sketches, and written notes) can be limited by perspective and subjectivity. 3D printing, combined with 3D scanning technology, allows for the creation of precise and detailed physical models of forensic evidence and crime scenes, as it can replicate the exact dimensions and spatial relationships of evidentiary material, providing an accurate representation for analysis [47]. Physical models enhance the visualization of complex scenes, making it easier for investigators, juries, and other stakeholders to understand the spatial dynamics and key elements [46, 48–50].

3D printed replicas offer solutions for preserving and analysing forensic evidence, such as bones, tools, and other physical objects. Forensic scientists can then conduct detailed examinations and experiments without damaging the original evidence. Replicas can be used for educational purposes, allowing trainees to handle and study exact copies of critical evidence such as the physical documentation and representation of child abuse [51]. In forensic anthropology, 3D printing has been instrumental in analyzing skeletal remains. It enables the creation of accurate models of bones and skulls, facilitating identification and trauma analysis. For example, a 3D print of a skull fracture can elucidate how the wounds were created by a unique blunt instrument, or used as a scaffold to reconstruct fragmentary remains [52, 53]. 3D printing aids in forensic facial reconstruction, where a digital model of a skull can be printed and used as a base for reconstructing the facial features of unidentified remains [40, 41].

The integration of 3D printing in forensic science also raises legal and ethical questions [54]. Courts must consider the admissibility of 3D printed models as evidence. 3D Printing can be a way to elucidate trauma and forensic evidence in a non-prejudicial way as opposed to bloody autopsy photos. 3D printing is transforming forensic science by providing new tools for crime scene reconstruction, evidence preservation, and analysis. Its applications in forensic anthropology and facial reconstruction have proven particularly valuable. However, addressing the legal, ethical, and technical challenges associated with this technology is crucial for its continued integration into forensic practices. A lot of work has been with respect to the acceptance of different modes of 3D data capture. The same cannot be said about 3D printing as it applies to forensics. In a recent systematic review with respect to 3D forensics, only 47 manuscripts were found with 3 discussing 3D printing for demonstration, 21 for practical applications, 8 examining methodological questions and 15 giving a general review [55]. In light of these findings, much more work is possible in establishing the application, reliability and usage of 3D printing in the field of forensics.

## Conclusion

For every forensic specialist dealing with imaging, it is important to always keep in mind the words of Wilhelm Conrad Roentgen, who said, “I did not think… I investigated.” [56]. This perfectly summarises the mindset that the forensic community should keep and develop, especially when it comes to the challenges of forensic imaging. In the history of medicine, post-mortem imaging is a very recent specialty, and it is still evolving both scientifically and practically. The scientific community, via the International Society of Forensic Radiology and Imaging, is becoming better organised year by year. It has been able to suggest recommendations and solid scientific bases on the contribution of post-mortem imaging to forensic practice, in both adults and children. There are going to be a lot of challenges over the next ten years. These include setting up national and international databases, looking into using artificial intelligence to read examinations and creating training courses that are based on effective and innovative teaching methods. As Rutty said, the forensic imaging team must keep an open mind [57].

## Data Availability

Non-applicable (review).

## Code availability

Non-applicable (review).

## References

[1] Daneshvari Berry S, Kroth PJ, Edgar HJH, Warner TD (2021) Developing the minimum dataset for the new Mexico decedent image database. Appl Clin Inf 12:518–527. 10.1055/s-0041-1730999 10.1055/s-0041-1730999 PMC8172257 34077973

[2] de Bakker HM, Soerdjbalie-Maikoe V, Kubat B et al (2016) Forensic imaging in legal medicine in the Netherlands: retrospective analysis of over 1700 cases in 15 years’ experience. J Forensic Radiol Imaging 6:1–7. 10.1016/j.jofri.2016.05.001

[3] Aghayev E, Staub L, Dirnhofer R et al (2008) Virtopsy– The concept of a centralized database in forensic medicine for analysis and comparison of radiological and autopsy data. J Forensic Leg Med 15:135–140. 10.1016/j.jflm.2007.07.005 18313007 10.1016/j.jflm.2007.07.005

[4] Esmaeilyfard R, Paknahad M, Dokohaki S (2021) Sex classification of first molar teeth in cone beam computed tomography images using data mining. Forensic Sci Int 318:110633. 10.1016/j.forsciint.2020.110633 33279763 10.1016/j.forsciint.2020.110633

[5] Zeng Y, Zhang X, Yoshizumi I et al (2023) Deep Learning-Based diagnosis of fatal hypothermia using Post-Mortem computed tomography. Tohoku J Exp Med 260:253–261. 10.1620/tjem.2023.J041 37197944 10.1620/tjem.2023.J041

[6] Zeng Y, Zhang X, Kawasumi Y et al (2023) A 2.5D deep Learning-Based method for drowning diagnosis using Post-Mortem computed tomography. IEEE J Biomed Health Inf 27:1026–1035. 10.1109/JBHI.2022.3225416 10.1109/JBHI.2022.3225416 36446008

[7] Pham CV, Lee S-J, Kim S-Y et al (2021) Age Estimation based on 3D post-mortem computed tomography images of mandible and femur using convolutional neural networks. PLoS ONE 16:e0251388. 10.1371/journal.pone.0251388 33979376 10.1371/journal.pone.0251388 PMC8115850

[8] Vila-Blanco N, Varas-Quintana P, Tomás I, Carreira MJ (2023) A systematic overview of dental methods for age assessment in living individuals: from traditional to artificial intelligence-based approaches. Int J Legal Med 137:1117–1146. 10.1007/s00414-023-02960-z 37055627 10.1007/s00414-023-02960-z PMC10247592

[9] Grassi L, Schileo E, Taddei F et al (2012) Accuracy of finite element predictions in sideways load configurations for the proximal human femur. J Biomech 45:394–399. 10.1016/j.jbiomech.2011.10.019 22079387 10.1016/j.jbiomech.2011.10.019

[10] Lotz JC, Cheal EJ, Hayes WC (1991) Fracture prediction for the proximal femur using finite element models: part I–Linear analysis. J Biomech Eng 113:353–360. 10.1115/1.2895412 1762430 10.1115/1.2895412

[11] Lotz JC, Cheal EJ, Hayes WC (1991) Fracture prediction for the proximal femur using finite element models: part II–Nonlinear analysis. J Biomech Eng 113:361–365. 10.1115/1.2895413 1762431 10.1115/1.2895413

[12] Lotz JC, Hayes WC (1990) The use of quantitative computed tomography to estimate risk of fracture of the hip from falls. J Bone Joint Surg Am 72:689–700 2355030

[13] Altai Z, Viceconti M, Offiah AC, Li X (2018) Investigating the mechanical response of paediatric bone under bending and torsion using finite element analysis. Biomech Model Mechanobiol 17:1001–1009. 10.1007/s10237-018-1008-9 29525976 10.1007/s10237-018-1008-9

[14] Raul J-S, Roth S, Ludes B, Willinger R (2008) Influence of the benign enlargement of the subarachnoid space on the bridging veins strain during a shaking event: a finite element study. Int J Legal Med 122:337–340. 10.1007/s00414-008-0242-6 18493785 10.1007/s00414-008-0242-6

[15] Raul J-S, Baumgartner D, Willinger R, Ludes B (2006) Finite element modelling of human head injuries caused by a fall. Int J Legal Med 120:212–218. 10.1007/s00414-005-0018-1 16059711 10.1007/s00414-005-0018-1

[16] Raul J-S, Deck C, Meyer F et al (2007) A finite element model investigation of gunshot injury. Int J Legal Med 121:143–146. 10.1007/s00414-005-0070-x 16362821 10.1007/s00414-005-0070-x

[17] Tuchtan L, Piercecchi-Marti M-D, Bartoli C et al (2015) Forces transmission to the skull in case of mandibular impact. Forensic Sci Int 252:22–28. 10.1016/j.forsciint.2015.04.017 25933425 10.1016/j.forsciint.2015.04.017

[18] LEVY AD, H JR (2020) Essentials of forensic imaging: a text-atlas. CRC, S.l

[19] Gorincour G (2020) Imagerie post mortem pratique et illustrée. Société française de radiologie, Paris

[20] Shenton A, Kralt P, Suvarna SK (2021) Post mortem CT for Non-Suspicious adult deaths: an introduction. Springer International Publishing, Cham

[21] Lo Re G, Argo A, Midiri M, Cattaneo C (2020) Radiology in forensic medicine: from identification to Post-mortem imaging. Springer International Publishing, Cham

[22] Dedouit F, Yen K, Heinze S (2022) Forensic imaging: A practical guide. Springer International Publishing, Cham

[23] Thali MJ, Viner MD, Brogdon BG (2011) Brogdon’s forensic radiology, 2nd edn. CRC, Boca Raton

[24] Tóth D, Petrus K, Heckmann V et al (2021) Application of photogrammetry in forensic pathology education of medical students in response to COVID-19. J Forensic Sci 66:1533–1537. 10.1111/1556-4029.14709 33764562 10.1111/1556-4029.14709 PMC8251483

[25] Paech D, Klopries K, Nawrotzki R et al (2022) Strengths and weaknesses of Non-enhanced and Contrast‐enhanced cadaver computed tomography scans in the teaching of gross anatomy in an integrated curriculum. Anat Sci Ed 15:143–154. 10.1002/ase.2034 10.1002/ase.2034 33170986

[26] Batko J, Chmiel R, Juszczak A et al (2023) The evaluation of vasculature in post-mortem angio-computed tomography for anatomy research purposes: method description based on Celiac trunk analysis. 10.5603/FM.a2023.0051. Folia Morphol VM/OJS/J/95676 10.5603/FM.a2023.0051 37622396

[27] Paech D, Klopries K, Doll S et al (2018) Contrast-enhanced cadaver-specific computed tomography in gross anatomy teaching. Eur Radiol 28:2838–2844. 10.1007/s00330-017-5271-4 29383525 10.1007/s00330-017-5271-4

[28] Brown W, Afshari S, Zhou M et al (2024) Living and post-mortem CT scans in the gross anatomy lab: A study investigating differences in first‐year medical students’ exam performance and perceptions. Anat Sci Ed Ase 2371. 10.1002/ase.2371 10.1002/ase.2371 38213130

[29] De Bakker BS, De Jong KH, Hagoort J et al (2016) An interactive three-dimensional digital atlas and quantitative database of human development. Science 354:aag0053. 10.1126/science.aag0053 27884980 10.1126/science.aag0053

[30] Chekrouni N, Kleipool RP, De Bakker BS (2020) The impact of using three-dimensional digital models of human embryos in the biomedical curriculum. Annals Anat - Anatomischer Anzeiger 227:151430. 10.1016/j.aanat.2019.151430 10.1016/j.aanat.2019.151430 31639440

[31] Carew RM, Errickson D (2019) Imaging in forensic science: five years on. J Forensic Radiol Imaging 16:24–33. 10.1016/j.jofri.2019.01.002

[32] Urbanová P, Jurda M, Vojtíšek T, Krajsa J (2017) Using drone-mounted cameras for on-site body documentation: 3D mapping and active survey. Forensic Sci Int 281:52–62. 10.1016/j.forsciint.2017.10.027 29101908 10.1016/j.forsciint.2017.10.027

[33] Ebert LC, Franckenberg S, Sieberth T et al (2021) A review of visualization techniques of post-mortem computed tomography data for forensic death investigations. Int J Legal Med 135:1855–1867. 10.1007/s00414-021-02581-4 33931808 10.1007/s00414-021-02581-4 PMC8354982

[34] Komar DA, Davy-Jow S, Decker SJ (2012) The use of a 3-D laser scanner to document ephemeral evidence at crime scenes and postmortem examinations. J Forensic Sci 57:188–191. 10.1111/j.1556-4029.2011.01915.x 21939441 10.1111/j.1556-4029.2011.01915.x

[35] Villa C, Lynnerup N, Jacobsen C (2023) A virtual, 3D multimodal approach to victim and crime scene reconstruction. Diagnostics (Basel) 13:2764. 10.3390/diagnostics13172764 37685302 10.3390/diagnostics13172764 PMC10486680

[36] Thompson TJU, Norris P (2018) A new method for the recovery and evidential comparison of footwear impressions using 3D structured light scanning. Sci Justice 58:237–243. 10.1016/j.scijus.2018.02.001 29685306 10.1016/j.scijus.2018.02.001

[37] Halim MIA et al (2024) Izyan Hani Imran, Adlina Syafura Ahmad Sabri, Forensic Investigation to Retrieve 3D Shoe Impression: A Review. ARASET 37:104–112. 10.37934/araset.37.2.104112

[38] Puentes K, Taveira F, Madureira AJ et al (2009) Three-dimensional reconstitution of bullet trajectory in gunshot wounds: a case report. J Forensic Leg Med 16:407–410. 10.1016/j.jflm.2009.04.003 19733332 10.1016/j.jflm.2009.04.003

[39] Chase CE, Liscio E (2023) Technical note: validation of Recon-3D, iPhone lidar for bullet trajectory Documentation. Forensic Sci Int 350:111787. 10.1016/j.forsciint.2023.111787 37481908 10.1016/j.forsciint.2023.111787

[40] Wilkinson C (2005) Computerized forensic facial reconstruction: A review of current systems. Forensic Sci Med Pathol 1:173–177. 10.1385/FSMP:1:3:173 25870042 10.1385/FSMP:1:3:173

[41] Decker S, Ford J, Davy-Jow S et al (2013) Who is this person? A comparison study of current three-dimensional facial approximation methods. Forensic Sci Int 229:161e1–161e8. 10.1016/j.forsciint.2013.03.028 10.1016/j.forsciint.2013.03.028 23628365

[42] Schweitzer W, Röhrich E, Schaepman M et al (2013) Aspects of 3D surface scanner performance for post-mortem skin Documentation in forensic medicine using rigid benchmark objects. J Forensic Radiol Imaging 1:167–175. 10.1016/j.jofri.2013.06.001

[43] Baiker M, Petraco NDK, Gambino C et al (2016) Virtual and simulated striated toolmarks for forensic applications. Forensic Sci Int 261:43–52. 10.1016/j.forsciint.2016.01.035 26874738 10.1016/j.forsciint.2016.01.035

[44] Fahrni S, Delémont O, Campana L, Grabherr S (2019) An exploratory study toward the contribution of 3D surface scanning for association of an injury with its causing instrument. Int J Legal Med 133:1167–1176. 10.1007/s00414-018-1973-7 30506239 10.1007/s00414-018-1973-7

[45] Karami A, Battisti R, Menna F, Remondino F (2022) 3D digitalization of transparent and glass surfaces: state of the Art and analysis of some methods. Int Arch Photogramm Remote Sens Spat Inf Sci XLIII-B 2–2022:695–702. 10.5194/isprs-archives-XLIII-B2-2022-695-2022

[46] Morris JM, Reichard RR, McGee KP (2022) 3D printing in forensic radiology. In: Wake N (ed) 3D printing for the radiologist. Elsevier, pp 157–173 10.1016/B978-0-323-77573-1.00004-X

[47] Katsioloudis P, Jones M (2015) Using Computer-Aided design software and 3D printers to improve Spatial visualization. Technol Eng Teach 74:14–20

[48] Simon G, Tóth D, Heckmann V, Poór VS (2022) Application of 3D printing in assessment and demonstration of stab injuries. Int J Legal Med 136:1431–1442. 10.1007/s00414-022-02846-6 35657431 10.1007/s00414-022-02846-6 PMC9375752

[49] Baier W, Warnett JM, Payne M, Williams MA (2018) Introducing 3D printed models as demonstrative evidence at criminal trials. J Forensic Sci 63:1298–1302. 10.1111/1556-4029.13700 29193075 10.1111/1556-4029.13700

[50] Henningsen MJ, Thorlacius-Ussing L, Jensen LG et al (2023) 3D printed skulls in court — a benefit to stakeholders? Int J Legal Med. 10.1007/s00414-023-03054-6 37391670 10.1007/s00414-023-03054-6 PMC10567900

[51] Barrera CA, Silvestro E, Calle-Toro JS et al (2019) Three-dimensional printed models of the rib cage in children with non-accidental injury as an effective visual-aid tool. Pediatr Radiol 49:965–970. 10.1007/s00247-019-04368-7 30877337 10.1007/s00247-019-04368-7

[52] Woźniak K, Rzepecka-Woźniak E, Moskała A et al (2012) Weapon identification using antemortem computed tomography with virtual 3D and rapid prototype modeling–a report in a case of blunt force head injury. Forensic Sci Int 222:e29–32. 10.1016/j.forsciint.2012.06.012 22748480 10.1016/j.forsciint.2012.06.012

[53] Jakobsen SR, Pedersen CC, Thomsen AH, Hansen K (2023) 3D-print as a template for reassembly of skull fragments in a homicide case. Annals 3D Print Med 12:100137. 10.1016/j.stlm.2023.100137

[54] Carew RM, French J, Morgan RM (2023) Drilling down into ethics: A thematic review of ethical considerations for the creation and use of 3D printed human remains in crime reconstruction. Sci Justice 63:330–342. 10.1016/j.scijus.2023.03.003 37169458 10.1016/j.scijus.2023.03.003

[55] Simon G, Poór VS (2022) Applications of 3D printing in forensic medicine and forensic pathology. A systematic review. Annals 3D Print Med 8:100083. 10.1016/j.stlm.2022.100083

[56] Thali MJ, Viner MD, Brogdon BG (2010) Brogdon’s forensic radiology, 0 edn. CRC

[57] Rutty GN (2023) Rutty’s rules: baseline guidance to safe postmortem computed tomography reporting. Forensic Imaging 34:200558. 10.1016/j.fri.2023.200558

